# Serum and Exosomal miR-7-1-5p and miR-223-3p as Possible Biomarkers for Parkinson’s Disease

**DOI:** 10.3390/biom13050865

**Published:** 2023-05-19

**Authors:** Lorenzo Agostino Citterio, Roberta Mancuso, Simone Agostini, Mario Meloni, Mario Clerici

**Affiliations:** 1 IRCCS Fondazione Don Carlo Gnocchi ONLUS, 20148 Milan, Italy; lcitterio@dongnocchi.it (L.A.C.); sagostini@dongnocchi.it (S.A.); mmeloni@dongnocchi.it (M.M.); mario.clerici@unimi.it (M.C.); 2 Department of Pathophysiology and Transplantation, University of Milan, 20100 Milan, Italy

**Keywords:** Parkinson’s disease, microRNA, α-synuclein, ddPCR, rehabilitation, inflammasome

## Abstract

The etiology of Parkinson’s disease (PD) is poorly understood, and is strongly suspected to include both genetic and environmental factors. In this context, it is essential to investigate possible biomarkers for both prognostic and diagnostic purposes. Several studies reported dysregulated microRNA expression in neurodegenerative disorders, including PD. Using ddPCR, we investigated the concentrations of miR-7-1-5p, miR-499-3p, miR-223-3p and miR-223-5p—miRNAs involved in the α-synuclein pathway and in inflammation—in the serum and serum-isolated exosomes of 45 PD patients and 49 age- and sex-matched healthy controls (HC). While miR-499-3p and miR-223-5p showed no differences (1), serum concentration of miR-7-1-5p was significantly increased (*p* = 0.0007 vs. HC) and (2) miR-223-3p serum (*p* = 0.0006) and exosome (*p* = 0.0002) concentrations were significantly increased. ROC curve analysis showed that miR-223-3p and miR-7-1-5p serum concentration discriminates between PD and HC (*p* = 0.0001, in both cases). Notably, in PD patients, both miR-223-3p serum (*p* = 0.0008) and exosome (*p* = 0.006) concentrations correlated with levodopa equivalent daily dosage (LEDD). Finally, serum α-synuclein was increased in PD patients compared to HC (*p* = 0.025), and in patients correlated with serum miR-7-1-5p in (*p* = 0.05). Our results suggest that both miR-7-1-5p and miR-223-3p, distinguishing PD from HC, have the potential to be useful and non-invasive biomarkers in Parkinson’s disease.

## 1. Introduction

Parkinson’s disease (PD) is one of the most common neurodegenerative diseases, second only to Alzheimer’s disease (AD), which mainly affects movement. The incidence rate of PD ranges from 5/100,000 to over 35/100,000 per year [1], and it especially affects the elderly population (2% of subjects over ages 65 and up to 4% of those over 85 years old) [2]. Clinically, the most common and recognized trademark motor symptoms of this disease are rigidity, postural instability, resting tremor and bradykinesia [3]. In addition, a wide range of non-motor symptoms, such as depression, hyposmia, sleep disturbance, constipation and dysautonomia, may occur in many patients prior to the onset of movement disorders typical of PD [4]. This disorder is characterized by the presence of protein accumulations of misfolded α-synuclein (α-Syn) called Lewy bodies, located in the substantia nigra of the brain [3], which contribute to the neuroinflammation observed in the disease [5]. Loss of dopaminergic neurons in the pars compacta of the substantia nigra leads to a reduction of voluntary movement ability [6]. PD is one of the complex polygenic disorders influenced by both genetic and environmental factors. In particular, variants located close to the *SNCA* gene, which encodes α-Syn, are strictly associated with the disorder [7,8]. Although *SNCA* is an extensively studied gene, the precise function of α-Syn and how it induces disease remains incompletely understood. PD etiology has been hypothesized to lie on a continuum ranging from monogenic inheritance to complex inheritance associated with the interplay between genetic risk and environmental influence [9]. The exact role of environmental factors in the pathogenesis of PD is only partially clarified. However, it is well established that both environmental and genetic factors correlate with cellular phenotypes that can be considered hallmarks of PD [6]. 

MicroRNAs (miRNAs) are a class of non-coding RNAs that play crucial roles as regulators of protein expression in several cell types, including neurons. By base-pairing with the 3′-untranslated regions (UTRs) of their target mRNAs, they inhibit translation into proteins or lead to mRNA degradation [10]. Aberrant expression of miRNAs has been observed in PD brains, suggesting that miRNAs that are known to regulate the expression of genes associated with neurodegeneration may contribute to the pathogenesis of PD [11]. These miRNAs have not only been detected in tissues, but also in body fluids including plasma and serum, urine, saliva, breast milk, seminal plasma and tears. They are produced within the nucleus, transported to the cytoplasm and then excreted into body fluids [12]. In recent years, miRNAs have also been identified in exosomes, i.e., extracellular vesicles that are produced by a variety of cells in eukaryotes and contain proteins, lipids, mRNA, and miRNAs. Exosomes are able to transport components originating from the cells in which they are produced and subsequently interact with other cells, allowing an exchange of information between different cells in both physiological and pathological conditions [13,14]. Recent studies have shown that endothelial cells take up neuron-derived exosomes, suggesting that neuronal exosomes and their cargo can cross the blood-brain barrier (BBB) and are capable of transferring their cargo to the periphery [15]. Exosomal miRNAs can exist stably in body fluids as they are not affected by ribonuclease (RNase) degradation [16]. Many studies have attempted to utilize serum exosomal miRNAs as biomarkers for neurodegenerative disease. In AD, it has been suggested that dysregulated miR-193b, together with miR-135a and miR-384, could be utilized as reliable biomarkers [17]. Similarly, downregulated miR-19b and upregulated miR-24 and miR-195 in serum exosomes are also considered to be diagnostic biomarkers for PD patients [18]. In addition, it has been previously observed that miR-223-3p in serum discriminates PD from healthy controls (HC), and also distinguishes AD from PD patients [19].

The primary aim of this work was to analyze the expression of serum and exosomal miRNAs in PD patients and HC in order to evaluate potential diagnostic biomarkers. In particular, we focused on three miRNAs known to be involved in the modulation of the *SNCA* gene—miR-223-3p, miR-7-1-5p, [20,21] and miR-499-3p, the latter targeting a specific sequence of α-Syn 3′UTR containing a PD-associated susceptibility polymorphism (rs17016074) [22]. Moreover, the evaluation of miR-7-1-5p and miR-223-3p is important: by targeting the *NLRP3* gene, we may be able to determine whether these miRNAs play a role in the neuroinflammation typically observed in PD [5]. Finally, a secondary aim of this study was to examine the concomitant expression in serum of miR-223-3p and its complementary strand miR-223-5p, as both miR-223 strands (-3p and -5p) are reported as deregulated in PD [19,23].

## 2. Materials and Methods

A total of 94 individuals were enrolled in the study: 45 PD patients (26 males and 19 females) and 49 sex- and age-matched HC (25 males and 24 females). All subjects were enrolled in rehabilitation programs and were followed by the Rehabilitative Neurology Unit of the IRCCS Santa Maria Nascente, Don C. Gnocchi Foundation–ONLUS (Milan, Italy). Patients were diagnosed as being affected by PD via clinical evaluation according to the Movement Disorder Society (MDS) Clinical Diagnostic Criteria for PD [24]. Levodopa equivalent daily dosage (LEDD) was calculated for each patient [25]. Neurological diseases were excluded in HC by clinical interview and examination. The study conformed to the ethical principles of the Declaration of Helsinki; all subjects gave informed and written consent according to a protocol approved by the local ethics committee of the Don Carlo Gnocchi Foundation (#06_21/06/2018). Serum was collected from all the enrolled subjects. The absence of serum hemolysis was confirmed by visual inspection and by the spectrophotometric measurement of hemoglobin absorbance at 414 nm [26].

Soluble total α-Syn (ng/mL) was measured in the serum of all the enrolled subjects via commercial Enzyme-Linked ImmunoSorbent Assays (ELISA) according to the manufacturer’s instructions (IBL International, Hamburg, Germany) [27]. 

Exosomes were isolated from serum samples using ExoQuick^®^ Exosome Isolation and RNA Purification Kit (SBI, Palo Alto, CA, USA), as previously reported [28], and re-suspended in 250 µL of sterile phosphate buffered saline (PBS). The miRNAs were extracted from serum as well from exosomes by QIAcube connect (Qiagen, Hilden, Germany) from 200 µL of serum using a spin column-based kit (miRNeasy Mini kit, Qiagen, Hilden, Germany). Quantification of miRNAs was performed using the Qubit 4 Fluorometer and Qubit microRNA Assay kit (Thermo Fisher, Foster City, CA, USA). The miRNAs were transcripted in complementary DNA (cDNA) by miRCURY LNA RT Kit (Qiagen) in a total volume of 10 µL. 

Quantification of miR-7-1-5p, miR-223-3p, miR-223-5p and miR-499-3p was performed by droplet digital PCR (ddPCR QX200, Bio-Rad, Hercules, CA, USA), whose assays were optimized by using serial dilutions of cDNA for each target, different primer concentrations and annealing temperatures. The ddPCR analysis was carried out using QuantaSoft software, version 1.7.4.0917 (Bio-Rad), and QX Manager, version 1.2 (Bio-Rad). In order to quantify copies of circulatory miRNAs, fluorescence intensity was employed to distinguish between negative and positive droplets. Positive controls as well as negative controls were included in each experiment. Thresholds were manually determined based on the negative controls, which included a no-template control. Only samples that produced more than two positive droplets and fell above a minimum amplitude threshold were considered positive. The detection limit is 0.8 copies/μL of reaction. Briefly, 3 µL of diluted cDNA (1:10) was mixed with miR-7-1-5p- and miR-499-3p-specific primers (Qiagen) and ddPCR EvaGreen Supermix (Bio-Rad). On the other hand, 5 µL of diluted cDNA (1:50) was used to quantify miR-223-3p. According to the manufacturer’s instructions, cDNAs were emulsified by a QX200 droplet generator, and the generated droplets were transferred to a 96-well reaction plate and heat-sealed with a pierceable sealing foil sheet (PX1, PCR plate sealer, Bio-Rad). PCR amplification was performed using a T100 thermal cycler (Bio-Rad) as follows: 10 min at 95 °C, 40 cycles at 94 °C for 30 s and at 58 °C for 60 s, followed by 10 min at 98 °C and a hold at 4 °C. The 96-well plate was then transferred to a QX200 droplet reader (Bio-Rad). Each well was queried for fluorescence to determine the number of positive events (droplets), and the results were displayed as dot plots. The miRNA concentration was expressed as copies/ng of extracted RNA.

Statistical analyses were performed using the commercial MedCalc Statistical Software package (Version 11.5.0.0; Ostend, Belgium). Normally distributed data were summarized as mean ± standard deviation, and the student *t*-test was employed to analyze the comparison among groups. On the other hand, not-normally distributed data were summarized as median and interquartile range (IQR: 25th and 75th percentile), using the Mann-Whitney test to analyze group comparisons. Correlations were analyzed using Spearman’s correlation coefficient, whereas receiver operating characteristics analysis (ROC) and area under curve (AUC) were used to evaluate the potential of miRNAs to be biomarkers. Qualitative data were compared using Fisher’s exact test and the Chi-squared test; the *p*-value was considered significant when ≤0.05.

## 3. Results

### 3.1. Demographic and Clinical Characterization of the Enrolled Population

Table 1 summarizes the demographic and clinical characteristics of PD patients and HC enrolled in the study. The populations were selected to be age- and sex-matched. Mean disease duration was 8.08 ± 4.9 years. Mean LEDD was 639.04 ± 243.53 mg/die and the median Hoehn and Yahr (H&Y) stage was 2 (IQR: 2–3; range 1–4).

### 3.2. Serum α-Syn Concentration

Serum concentration of α-Syn was significantly increased in PD (19.26 ng/mL; 11.80–25.13 ng/mL) compared to HC (14.62 ng/mL; 8.76–23.32 ng/mL; *p* = 0.025). No correlations were found in PD between α-Syn serum concentration and either age, gender, LEDD or H&Y score.

### 3.3. Serum and Exosomal miR-7-1-5p Concentration by Droplet Digital PCR (ddPCR)

**Figure 1:**
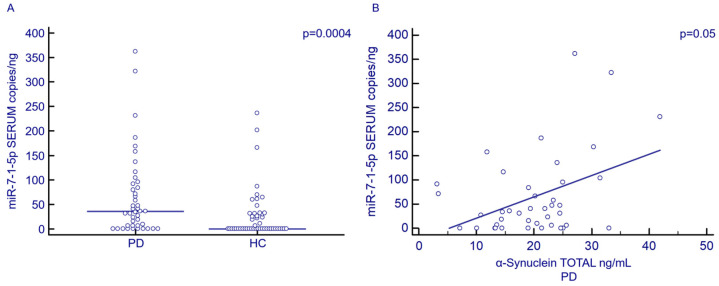
Serum miR-7-1-5p concentration (copies/ng) in PD patients (N = 45) and HC (N = 49) (**A**). Correlation between serum miR-7-1-5p concentration (copies/ng) and α-Syn (ng/mL) in PD patients (**B**).

Serum concentration of miR-7-1-5p was significantly increased in PD (35.54 copies/ng; 5.82–89.61 copies/ng) compared to HC (0.00 copies/ng; 0.00–30.97 copies/ng; *p* = 0.0007) (Figure 1A). No statistically significant differences were observed when miR-7-1-5p exosomal concentration was analyzed. Receiver Operating Characteristics (ROC) curve analysis showed that serum miR-7-1-5p concentration discriminates between PD and HC (*p* = 0.0001, AUC: 0.7; 95%CI: 0.6–0.8; sensibility: 54.5%; specificity: 81.6%). Notably, a correlation was detected between the serum concentration of miR-7-1-5p and α-Syn in PD patients alone (*p* = 0.05) (Figure 1B). Finally, no correlations were observed between serum or exosomal miR-7-1-5p concentration and either gender, H&Y stage or disease duration.

### 3.4. Serum and Exosomal miR-223-3p Concentration by ddPCR

**Figure 2:**
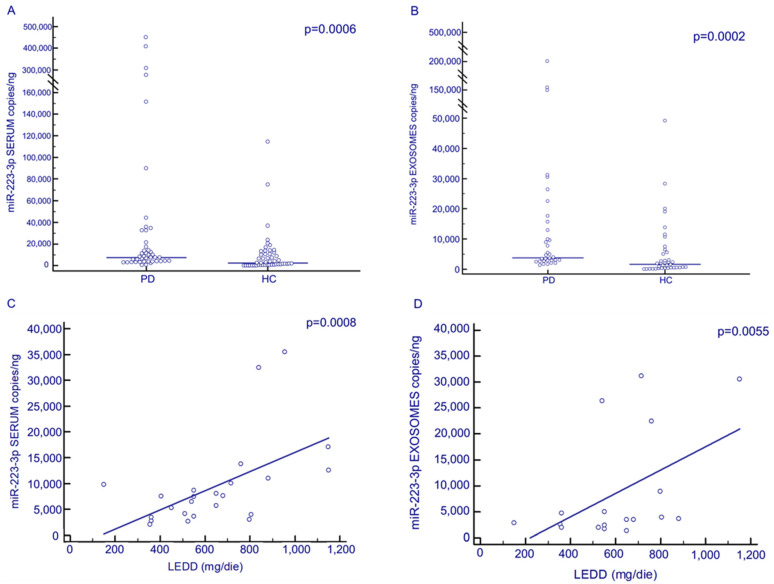
Above, serum (**A**) and exosomal (**B**) miR-223-3p expression (copies/ng) in PD patients (N = 45) and HC subjects (N = 49). Below, correlation between serum (**C**) and exosomal (**D**) miR-223-3p expression with levodopa equivalent daily dosage (LEDD, mg/die).

The concentration of miR-223-3p was significantly increased in both the serum (PD: 7843.02; 3761.14–26,972.84 copies/ng; HC: 2593.12 copies/ng; 502.96–10,989.89 copies/ng; *p* = 0.0006) and exosomes (PD: 4715.67 copies/ng; 2832.13–16,175.03 copies/ng; HC: 1698.53 copies/ng; 463.20–5547.85 copies/ng; *p* = 0.0002) of PD patients compared to HC (Figure 2A,B). ROC curve analysis showed that miR-223-3p serum concentration discriminates between PD and HC (*p* = 0.0001, AUC: 0.7; 95%CI: 0.6–0.8; sensibility: 93.2%; specificity: 51.2%). Interestingly, both serum and exosomal miR-223-3p concentrations were significantly correlated with LEDD (serum: *p* = 0.0008; exosomes: *p* = 0.04) (Figure 2C,D). Finally, no correlations could be detected between miR-223-3p and either gender, age, H&Y stage, disease duration or α-Syn serum concentration.

### 3.5. Analyses of the Prediction of the Disease with Combined Serum -miR-223-3p and -miR-7-1-5p

**Figure 3:**
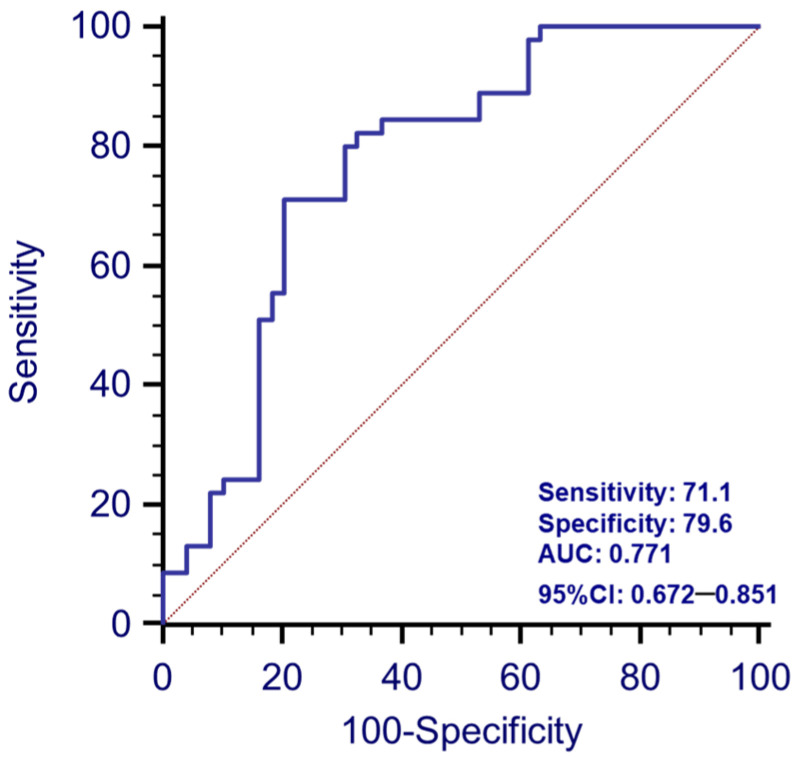
ROC analysis: Receiver Operating Characteristic (ROC) curve analysis showed the discriminative power of the two-variable combination as the product of the two values (serum-miR-223-3p and -miR-7-1-5p) in the enrolled population.

In performing the ROC curve analysis with the combination of the two miRNAs (product of the two values obtained in serum), we found a sensitivity of 71.1, a specificity of 79.9 and, notably, an AUC value of 0.771 (*p* < 0.0001), which was higher than the one provided by the previous two ROC curve analyses with the two separate miRNAs (0.71 for both) (Figure 3). Furthermore, in performing the Chi-squared test, we found that only in 2/45 (4%) PD patients was the expression of both miR-7-1-5p and miR-223-3p below the cut-off threshold derived from the respective ROC curves (*p* < 0.0001). Finally, both miRNAs were expressed above the cut-off threshold in only 6/49 (12%) of HC (*p* < 0.0001).

### 3.6. Serum and Exosomal miR-499 and miR-223-5p Concentration by ddPCR

Both miR-499-3p and miR-223-5p were detectable in the serum of only a portion of individuals (44% and 59%, respectively), and no statistical differences were found between PD and HC. These two miRNAs could not be measured in exosomes of either PD or HC.

## 4. Discussion

The aim of this study was to evaluate whether circulatory miRNAs that bind α-Syn or are involved in neuroinflammation could be possible biomarkers for PD.

The most significant findings of the present study are that serum expression of miR-7-1-5p and both serum and exosomal expression of miR-223-3p are increased in PD patients compared to HC. To note, the efficacy of these two miRNAs as biomarkers for PD, analyzed by ROC curve, is higher when their serum concentrations were evaluated together versus when they were considered alone. Importantly, Horst and coworkers [29] have found that miR-7-1-5p is less expressed in the brain tissues of PD patients compared to HC. Moreover, recent studies showed that miR-7-5p has two specific binding sites with 3′UTR of α-Syn mRNA, and its overexpression could reduce *SNCA* transcription [30]. Since α-Syn aggregates in the brain are a hallmark of PD, and considering that miR-7-1-5p targets the 3’UTR region of *SNCA* mRNA, upregulation of this miRNA in the blood could be an attempt to reduce the excessive production of the α-Syn protein, as was previously suggested [31]. Other studies will be necessary to verify these hypotheses in order to shed light on the role of miR-7-1-5p in blood.

Additionally, miR-7 targets other mRNAs coded by genes that contribute to proliferation of neural stem cells, repair of peripheral nerve injures and neuronal homeostasis, potentially modulating processes such as oxidative stress, mitochondrial health, glycolysis, apoptosis, inflammasome activation and cancer proliferation [32,33,34,35,36,37,38]. The molecular interaction between miR-7 and *NLRP3* has been suggested to play a role in the pathogenesis of PD, as a reduced expression of miR-7-5p seems to result in upregulation of *NLRP3*, directly or indirectly, via a α-Syn-mediated activation of the *NLRP3* inflammasome [37]. Very few data have investigated serum miR-7-5p in PD patients. Bai and coworkers [39] showed that concentrations of this molecule are slightly increased in PD patients, without reaching statistical significance, which is possibly the consequence of the fact that analyses were performed with a traditional qPCR as opposed to the innovative and more sensitive ddPCR that we have used in our work. Older results [37] found a lower concentration of miR-7-5p in the serum of PD patients compared to HC. Very few patients were enrolled in that study, and results stem from analyses performed using a traditional qPCR and the endogenous U6 as housekeeping gene, a method not universally accepted [40,41]. 

Serum concentration of miR-223-3p was also increased in PD patients, confirming recent data [19]. miR-223-3p is a crucial miRNA in PD because, besides to binding α-Syn, this miRNA plays a pivotal role in the modulation of the inflammasome, acting as a negative regulator of *NLRP3* expression [42]. In addition, miR-223-3p is involved in other molecular pathways that are related to the pathogenesis of PD, including calcium fluxes [43], the NF-kB pathway [44], cellular differentiation and proliferation [45], the pathogenesis of dementia [46] and cancers [47]. For these reasons, a better understanding of the mechanisms responsible for miR-223-3p upregulation in PD patients could be important for shedding light on the molecular mechanisms associated with this disease. 

Notably, we measured these two miRNAs in serum-isolated exosomes, very small vesicles that can derive from the central nervous system (CNS) and reach the periphery as well [15]. In this work, we included the analysis of miRNA expression in exosomes, considering their ability to pass through the BBB. Among the molecules carried by exosomes, there are also miRNAs, whose altered expression may be indicative of an ongoing inflammatory process or tissue damage in the CNS caused by neurodegeneration. In addition, miR-223-3p was found to be significantly up-regulated in exosomes of PD patients, confirming the results obtained from serum, whereas the exosome content of miR-7-1-5p was similar in PD and HC. This result was quite unexpected, and suggests that circulating miR-7-1-5p is mainly present as free or protein-associated and not exosome-associated. This needs to be investigated more extensively, but could this be also due to the relatively low expression of miR-7-1-5p compared to miR-223-3p.

Interestingly, miR-223-3p concentration in both serum and exosomes was positively correlated with LEDD. Levodopa crosses the BBB, binds the DOPA decarboxylase inhibitor (or carbidopa) and is converted into active dopamine [48]: the most effective therapy for PD [49]. Since patients with severe symptoms usually receive higher concentrations of LEDD, the positive association between this parameter and miR-223-3p indicates that serum and exosomal concentrations of this miRNA might be related to disease severity, although no correlation has been observed between H&Y and miR-223-3p concentration. However, analyses focusing on patients with very mild symptoms, compared to those with severe disease, will be needed in order to confirm this hypothesis, as the majority of our study population showed a H&Y score close to 2. Moreover, as dopamine seems to be a regulator of *NLRP3* activation in macrophages [50], studying the effect of different DOPA concentrations on the lymphocytes of PD patients will be of interest to verify the possible interaction among this drug, *NLRP3* and miR-223-3p. Of note, Cressatti et al. [51] found no correlation between salivary miR-223-3p and LEDD, but, to our knowledge, this is the first study that found a correlation between serum miR-223-3p and LEDD in PD patients. These data are intriguing and will need to be validated by further investigations.

Finally, whereas the direct correlation between α-Syn and miR-7-1-5p indicates that miR-7-1-5p production could be associated with the impairment in α-Syn metabolism that characterizes PD [29,52], the absence of correlation between miR-223-3p and α-Syn suggests that the concentration of this miRNA is dependent on other pathogenic mechanisms involved in PD, possibly including neuroinflammation [53].

The potential of α-Syn as a biomarker in PD has been extensively investigated due to its involvement in the disease and its presence in cerebrospinal fluid (CSF), blood and other bodily fluids [54]. We found higher levels of total α-Syn in PD serum samples compared to HC. The accumulation of α-Syn in the CNS is a trademark of PD, and recent evidence shows that this protein is expressed also in peripheral tissues [55]. Our results are consistent with recent results showing that α-Syn is significantly increased in PD serum, and its concentration is correlated with the degree of symptoms present in early-stage PD patients [56,57]. However, it is important to underline that discordant results are reported for serum concentration in patients [58,59]. This discrepancy could be explained by considering that blood cells, including erythrocytes and platelets, contain high concentrations of α-Syn, and in case of hemolysis or contamination, the concentration of this protein in serum would be altered [60]. Moreover, measuring α-Syn is tricky, as this protein can exist in multiple monomeric and oligomeric forms, as well as in post-translational modified forms [59]. Finally, no differences regarding miR-223-5p and miR-499-3p circulatory concentrations were observed between PD and HC.

In conclusion, although further studies will be necessary to confirm these preliminary findings, i.e., measurement of Ca+ flux and NLRP3 mRNA in blood cells, results herein suggest that the combined measurement of circulatory miR-223-3p, miR-7-1-5p, together with an increased concentration of α-Syn, could discriminate between PD patients and HC, possibly indicating these three molecules are useful biomarkers in Parkinson’s disease.

## Figures and Tables

**Table 1:** Demographic and clinical characteristics of the enrolled populations.

Demographic and Clinical Characteristics	PD Patients	Healthy Controls
N	45	49
Gender (M-F)	26–19	25–24
Age, years (means ± SD)	67.30 ± 9.02	65.49 ± 12.15
Disease duration, years (mean ± SD)	8.08 ± 4.90	-
LEDD, mg/die (mean ± SD)	639.04 ± 243.53	-
H&Y stages (median; IQR)	2; 2–3	-
α-Syn in serum, ng/mL (median; IQR)	19.26; 11.80–25.13	14.62; 8.76–23.32

PD: Parkinson’s disease; M: male; F: female; N: absolute number; SD: standard deviation; IQR: interquartile range; LEDD: levodopa equivalent daily dosage; H&Y: Hoehn and Yahr scale.

## Data Availability

The data presented in this study are available on request from the corresponding author.
